# 2000. Identifying Limitations and Opportunities for Undergraduate and Medical Student Involvement in Infectious Diseases Nationwide

**DOI:** 10.1093/ofid/ofad500.127

**Published:** 2023-11-27

**Authors:** Divyam Goel, Michelle Tin, Krishna C Hariprasad, Diya S Garg, Arnel Besic, Tilly A Dillon, Zoe R Masson, Lauren Goralsky, Julia A Goralsky, Molly K Barron, Jasmine A Saji, Wendy L Hobson-Rohrer, Trahern Wallace. Jones

**Affiliations:** University of Utah, Holladay, UT; University of Utah, Holladay, UT; University of Texas at Austin, Austin, Texas; University of Utah, Holladay, UT; University of Utah, Holladay, UT; University of Utah, Holladay, UT; University of Utah, Holladay, UT; Columbia University, New York, New York; Columbia University, New York, New York; Temple University, Philadelphia, Pennsylvania; Illinois Institute of Technology, Mount Prospect, Illinois; University of Utah, Holladay, UT; University of Utah, Holladay, UT

## Abstract

**Background:**

Only 74% of infectious disease (ID) training positions were filled in the 2022 fellowship match. Novel and creative approaches to increasing interest and recruitment in the field must be explored. Here, we identify the lack of ID representation in medically-related student interest groups and clubs at the undergraduate and medical school levels nationwide and suggest such groups be considered for recruitment strategies.

**Methods:**

The websites of 2,321 Universities and 173 medical schools across the United States were manually searched for the presence of undergraduate clubs and interest groups, respectively, for multiple medical specialties and subspecialties, including ID. Fields considered included ID, microbiology, emergency medicine, global/public health, neuroscience/psychology, pre-health, and STEM at the undergraduate level, and ID, wilderness medicine, global health, hematology/oncology, radiology, dermatology, family medicine, internal medicine, orthopedics/sports medicine, neurology/psychiatry, and emergency medicine at the medical school level. Furthermore, zip code data from mailing addresses was used to compare the proximity of 165 adult ID Fellowships to undergraduate institutions.

Inclusion and Exclusion Criteria for Undergraduate Institutions

**Figure:**
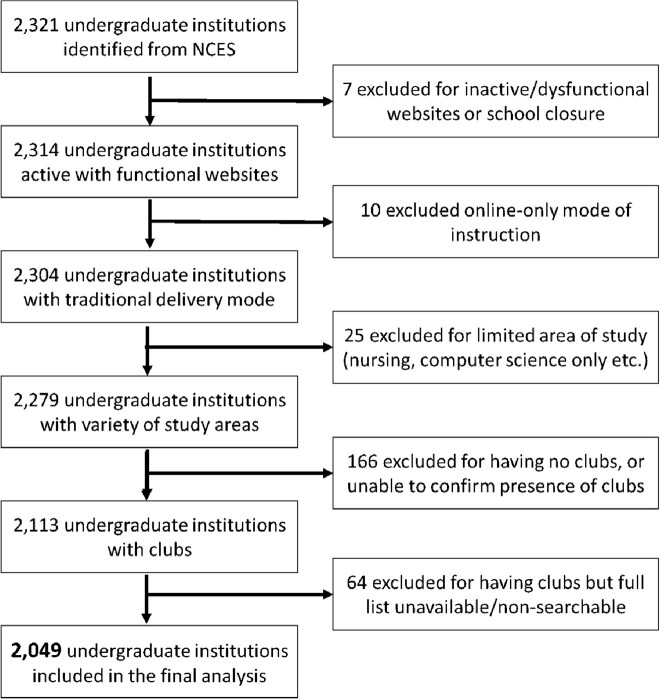

Criteria were designed to exclude institutions unlikely to serve students with an eventual goal of pursuing medicine.

**Results:**

Of 2,049 undergraduate institutions meeting inclusion criteria nationwide, 6 (0.29%) had a club for ID. Of 163 medical schools meeting inclusion criteria, 57 (35.0%) had an interest group for ID. ID student groups were the least prevalent among all the categories considered at both the undergraduate and medical school level. Our geographic proximity analysis found that every adult ID fellowship is in the same city and/or county as at least one undergraduate institution, and 28.5% of adult ID fellowships are in the same zip code as at least one undergraduate institution

**Figure:**
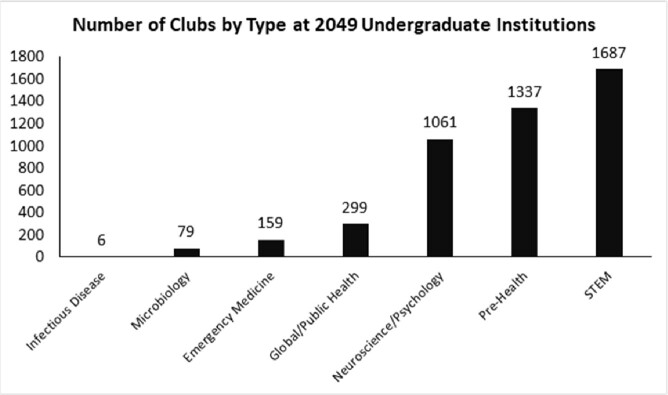

The websites of 2,321 undergraduate institutions with a minimum of 1,000 undergraduate enrollments were manually searched for the presence of medically-related clubs in various fields, including ID. Club counts are shown for 2,049 institutions meeting inclusion criteria.

**Figure:**
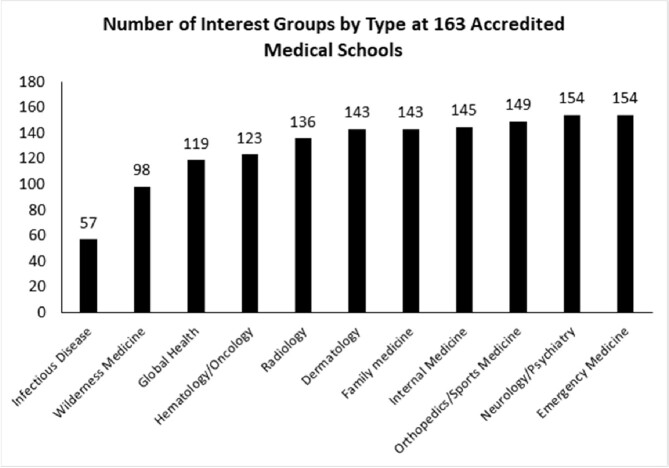

The websites of 173 medical schools were manually searched for the presence of medically-related interest groups in various fields, including ID. Group counts are shown for the 163 institutions with full accreditation status.

**Conclusion:**

ID clubs and interest groups are the least prevalent among medically-related student interest groups. This paucity presents an opportunity for the ID community to begin outreach and recruitment at the undergraduate and medical student levels, specifically through student groups. Our own group, InfectED, can be used as a model for future groups nationwide.

**Disclosures:**

**All Authors**: No reported disclosures

